# Fetal Inflammatory Response Syndrome and Cerebral Oxygenation During Immediate Postnatal Transition in Preterm Neonates

**DOI:** 10.3389/fped.2020.00401

**Published:** 2020-07-22

**Authors:** Christina Helene Wolfsberger, Marlies Bruckner, Nariae Baik-Schneditz, Bernhard Schwaberger, Lukas Peter Mileder, Alexander Avian, Berndt Urlesberger, Gerhard Pichler

**Affiliations:** ^1^Division of Neonatology, Department of Pediatrics and Adolescent Medicine, Medical University of Graz, Graz, Austria; ^2^Research Unit for Neonatal Micro- and Macrocirculation, Department of Pediatrics and Adolescent Medicine, Medical University of Graz, Graz, Austria; ^3^Institute for Medical Informatics, Statistics and Documentation, Medical University of Graz, Graz, Austria

**Keywords:** preterm neonates, fetal inflammatory response syndrome (FIRS), inflammation, interleukin-6, cerebral oxygenation, near-infrared spectroscopy (NIRS), transition period

## Abstract

**Introduction:** Fetal inflammatory response syndrome (FIRS), defined as elevated umbilical cord blood interleukin-6 (IL-6) values > 11 pg/ml, is associated with an increased risk of neonatal morbidity and mortality. The primary aim of the present study was to evaluate a potential influence of FIRS on cerebral oxygen saturation (crSO2) and fractional tissue oxygen extraction (cFTOE) during immediate postnatal transition in preterm neonates. The secondary aim was to analyze the potential influence of FIRS on cerebral injury and mortality.

**Methods:** Secondary outcome parameters of prospective observational studies were analyzed. Preterm neonates with measured IL-6 values from umbilical cord blood and cerebral near-infrared spectroscopy (NIRS) measurements during immediate transition after birth were included. Preterm neonates with FIRS (FIRS group) were matched 1:1 for gestational age (± 1 week) to preterm neonates without FIRS (non-FIRS group). crSO2, cFTOE, arterial oxygen saturation (SpO2), heart rate (HR), and fraction of inspired oxygen (FiO2) were compared between both groups. In addition, cerebral injury and mortality were compared between both groups.

**Results:** A total of 46 preterm neonates were included. Twenty-three neonates in the FIRS group [median gestational age 32.1 (IQR 30.3–33.0) weeks; median IL-6 19.7 (IQR 12.2–37.0) pg/ml] were compared to 23 neonates in the non-FIRS group [gestational age: 32.0 (30.4–33.1) weeks; IL-6: 5.4 (3.0–6.7) pg/ml]. cFTOE showed significantly lower values within the first 4 min and a trend toward lower values in minute 5 after birth in the FIRS group. There were no significant differences in crSO2 within the first 15 min after birth between the two groups. SpO2 was significantly lower in minutes 5 and 6 and HR was significantly lower in minutes 2 and 4 after birth in the FIRS group compared to the non-FIRS group. Survival without cerebral injury was similar in both groups.

**Conclusion:** In preterm neonates with FIRS the crSO2 was similar despite significantly lower cFTOE values during the first minutes after birth. This observation may be a result of compromised oxygen consumption and delivery in the first minutes after birth in neonates with FIRS.

## Introduction

Fetal inflammatory response syndrome (FIRS) is defined as elevated interleukin-6 (IL-6) values in umbilical cord blood (IL-6 > 11 pg/ml) (1). FIRS is a condition of systemic activation of the fetal immune system and is associated with a higher risk of neonatal morbidity and mortality (1–3). Originally, FIRS was described as elevated fetal plasma IL-6 values in fetuses of mothers with preterm premature rupture of membranes (1). Preterm neonates born with FIRS show a higher prevalence of infant respiratory distress syndrome, sepsis, intraventricular hemorrhage (IVH), periventricular leukomalacia (PVL), bronchopulmonary dysplasia, cerebral palsy, and death (1, 4–10).

In FIRS, pro- and anti-inflammatory cytokine release leads to oxidative stress resulting in cerebral cell damage (11). IL-6 is already known to be a risk factor of white matter injury (12). This raises the question if FIRS is associated with a compromised cerebral tissue oxygen saturation (crSO2) in neonates, aggravating adverse effects and cerebral injury.

Near-infrared spectroscopy (NIRS) enables non-invasive, continuous measurement of crSO2 and cerebral tissue fractional oxygen extraction (cFTOE) (13–15). Rallis et al. (16) observed in septic neonates a decrease in cerebral oxygenation (measured with NIRS) over the first seven days after birth. Cerebral NIRS monitoring is well established in the delivery room during the immediate transition period from intra- to extrauterine life (17–20). However, so far there are no data about the effect of FIRS on crSO2 during the immediate transition after birth in preterm neonates.

The primary aim of the present study was to evaluate whether there is an association between FIRS and crSO2/cFTOE in preterm neonates during the first 15 min after birth. We hypothesized that in preterm neonates with FIRS, crSO2 values are lower and cFTOE is higher due to compromised perfusion. In addition, our secondary aim was to analyze cerebral injury and mortality until term-equivalent age or before discharge in neonates with and without FIRS.

## Methods

### Design

In the present study secondary outcome parameters of prospective observational studies, conducted between May 2010 and November 2019 at the Division of Neonatology, Medical University of Graz, Austria, were analyzed. All studies were approved by the Regional Committee on Biomedical Research Ethics (EC numbers: 19/291 ex 07/08, 23/403 ex 10/11, 27–465 ex 14/15, 30–450 ex 17/18) and written parental consent was obtained before study inclusion.

### Patients

We included preterm neonates, in whom umbilical cord blood IL-6 values and crSO2 were measured during the first 15 min after birth. Exclusion criteria were major congenital anomalies. The preterm neonates were stratified into two groups according to their IL-6 values: neonates with IL-6 ≤ 11 pg/ml were assigned to the non-FIRS group, and those with IL-6 > 11 pg/ml to the FIRS group (1).

### NIRS and Routine Monitoring

An INVOS 5100C Cerebral/Somatic Oximeter Monitor (Covidien, Massachusetts, U.S.A.) with a neonatal transducer was used for crSO2 measurements. After birth the cord was clamped according routine after at least 30 s. Preterm neonates were placed on the resuscitation table under an overhead heater immediately after birth. The NIRS transducer was applied on the left fronto-parietal head in each neonate immediately after birth without disturbing routine medical care. The sensor was secured with a gauze bandage. crSO2 measurements were conducted during the first 15 min after birth. The sample rate (period) of NIRS measurements was 0.13 Hz (8 s).

Arterial oxygen saturation (SpO2) and heart rate (HR) were measured with the IntelliVue MP30 monitor (Philips, The Netherlands). The transducer was placed on the right hand/wrist.

crSO2, SpO2 and HR were recorded continuously during the first 15 min after birth and stored every second in a multichannel system (alpha-trace digital MM, B.E.S.T. Medical Systems, Austria) for subsequent analyses. cFTOE was calculated for each minute: (SpO2-crSO2)/SpO2.

Body temperature was measured in minute 15 after birth using a rectal probe. Blood pressure was measured by a pneumatic cuff applied to the right upper arm with the IntelliVue MP30 monitor (Philips, The Netherlands) in minutes 5, 10, and 15 after birth. Afterwards, the mean of the three mean arterial blood pressure (MABP) values was calculated. Further, fraction of inspired oxygen (FiO2) was recorded and the need for respiratory support (continuous positive airway pressure (CPAP) or intubation) was documented.

### IL-6, Procalcitonin and C-Reactive Protein

IL-6 was measured in umbilical cord blood plasma taken immediately after birth. The analysis of IL-6 was performed using the Endogen Interleukin-6 ELISA (Endogen Inc., Massachusetts, U.S.A.) according to the standard procedure. In addition, procalcitonin (PCT) levels were analyzed from the same umbilical cord blood sample. C-reactive protein (CRP) was determined twice after birth: within 24–48 h and 48–72 h after birth.

### Cerebral Injury and Mortality

Cerebral injuries were evaluated by routine cerebral ultrasound examinations carried out in all preterm neonates on the first, fourth, and eighth day after birth and at term age or before discharge, depending on what came first. We recorded any grade of IVH and PVL, and death.

### Groups Matching

Preterm neonates in the non-FIRS group were matched for gestational age ± 1 week to those of the FIRS group. The matching ratio was 1:1.

### Statistical Analysis

Data are presented as mean and standard deviation or median and interquartile range (IQR) for continuous data and absolute and relative frequency for categorical data, respectively. Baseline differences between groups were analyzed using *t*-test or Mann-Whitney U test for continuous data and Chi-square test or Fishers's exact test for categorical data. A linear mixed model with fixed effect for time and first-order autoregressive covariance structure was used for calculation of overall effects and differences between groups at each minute. The course of parameters was analyzed starting with the second minute after birth until minute 15. The first minute of life was not analyzed due to the high number of missing values. For the visualization of the courses of analyzed parameter estimated values according to the linear mixed model with 95% confidence intervals (95%CI) are shown. Lower bound of the 95%CI for FiO2 were bounded to a minimum of 21%, since lower values are not physiological. A *p*-value of *p* < 0.05 was considered statistically significant. Statistical analyses were performed using SPSS 26.0 (SPSS, Chicago, IL, USA).

## Results

**Figure 1:**
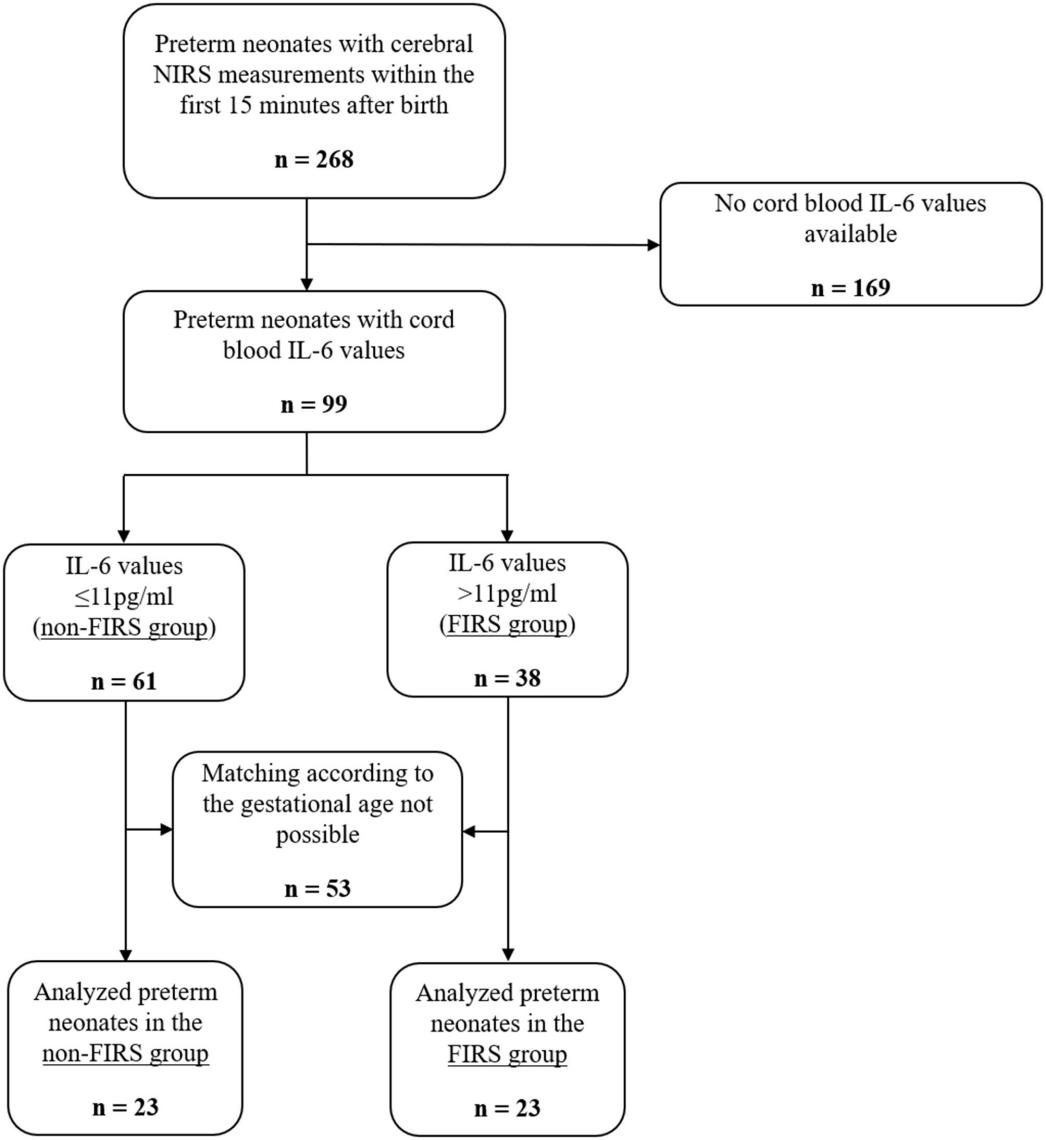
Study flow chart showing the number of included and analyzed preterm neonates and rationales for exclusion.

Two-hundred sixty-eight preterm neonates were included in the prospective observational studies. Ninety-nine preterm neonates, with IL-6 values available and crSO2 measurements within the first 15 min after birth, fulfilled the inclusion criteria. Sixty-one neonates showed IL-6 values ≤ 11 pg/ml (non-FIRS group) and 38 neonates had IL-6 values > 11 pg/ml (FIRS group). Twenty-three neonates in each group, matched for gestational age, were finally analyzed (Figure 1). Demographic data are presented in Table 1.

**Table 1:** Demographic data and routine monitoring parameters in 23 preterm neonates with FIRS and 23 preterm neonates without FIRS (FIRS group and non-FIRS group).

	**FIRS**	**non-FIRS**	***p*-value**
*n*	23	23	
Gestational age (weeks)	32.1 (30.3–33.0)	32.0 (30.4–33.1)	0.895
Birth weight (g)	1,670 (1,214–1,958)	1,630 (1,400–1,994)	0.965
Female sex	12 (52)	13 (57)	0.767
Delivery by cesarean section	19 (83)	23 (100)	0.109
Umbilical artery pH	7.31 (7.27–7.34)	7.33 (7.30–7.33)	0.485
Mean arterial blood pressure (mmHg)	38 ± 6	43 ± 12	0.154
Rectal body temperature (°C)	36.5 ± 0.4	36.9 ± 0.4	0.007^*^
Apgar 1 min	8 (7–9)	8 (8, 9)	0.930
Apgar 5 min	9 (8, 9)	9 (8, 9)	0.364
Apgar 10 min	9 (9, 10)	9 (9)	0.828
Interleukin-6 (pg/ml)	19.7 (12.2–37.0)	5.4 (3.0–6.7)	<0.001^*^
Procalcitonin (ng/ml)	0.17 (0.12–0.26)	0.19 (0.15–0.23)	0.426
C-reactive protein 24–48 h after birth (mg/dl)	0.65 (0.60–2.80)	0.90 (0.60–2.30)	0.570
C-reactive protein 48–72 h after birth (mg/dl)	1.30 (0.60–3.80)	0.60 (0.60–1.90)	0.531

*Data are presented as mean ± SD, median (IQR) or n (%)*.

* *p-value < 0.05*.

Indication for preterm birth were preterm labor (FIRS group *n* = 4/non-FIRS group *n* = 5), preterm premature rupture of membranes (*n* = 6/*n* = 3), preeclampsia/HELLP syndrome/maternal hypertension (*n* = 4/*n* = 6), intrauterine growth restriction (*n* = 2/*n* = 3), and other reasons (*n* = 7/*n* = 6). Nineteen (83%) preterm neonates were delivered by cesarean section in the FIRS group and 23 (100%) neonates in the non-FIRS group.

Twenty (87%) preterm neonates in the FIRS group and 21 (91%) preterm neonates in the non-FIRS group needed respiratory support and/or supplemental oxygen (CPAP) within the first 15 min after birth. Four (17%) neonates in the FIRS group and one (4%) neonate in the non-FIRS group were intubated within the first 15 min after birth. None of the neonates needed cardiopulmonary resuscitation.

### NIRS Monitoring

**Figure 2:**
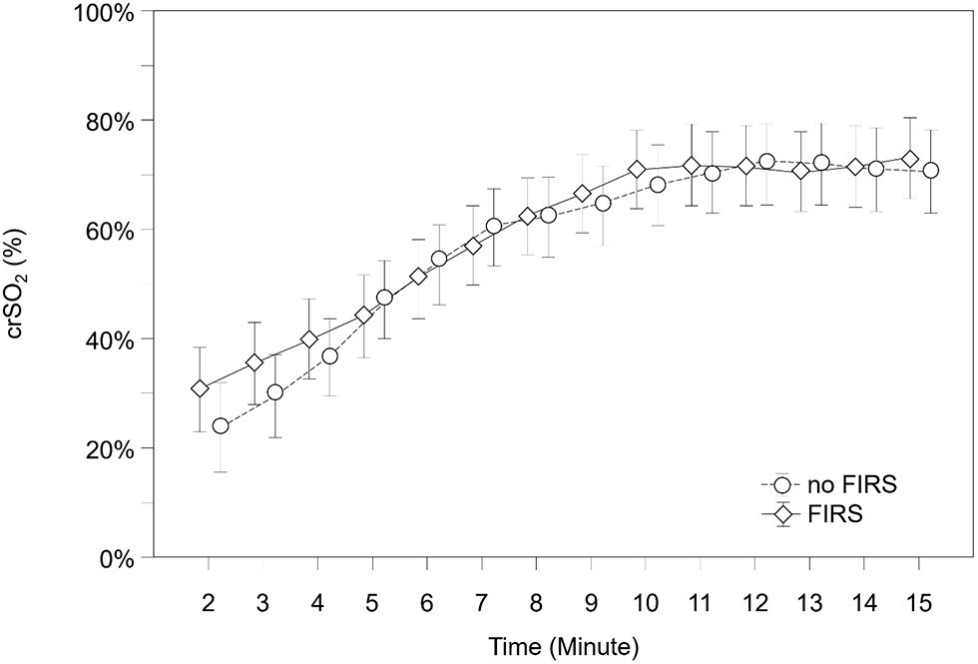
crSO2 in 23 preterm neonates with FIRS and 23 preterm neonates without FIRS (FIRS group and non-FIRS group) during the first 15 min after birth (mean and 95% CI).

**Figure 3:**
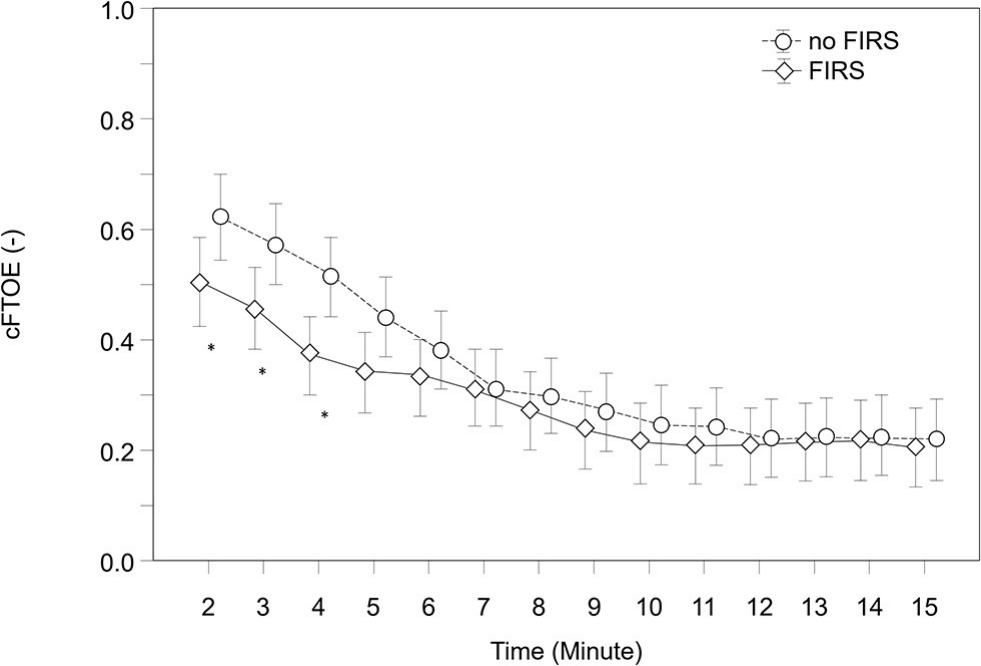
cFTOE, in 23 preterm neonates with FIRS and 23 preterm neonates without FIRS (FIRS group and non-FIRS group) during the first 15 min after birth (mean and 95% CI) (*outliers).

The courses of crSO2 and cFTOE during the first 15 min after birth are demonstrated in Tables 2, 3 and Figures 2, 3. There were no significant differences in crSO2 between the two groups. In the FIRS group, cFTOE was significantly lower in minutes 2, 3, and 4, and showed a trend toward a lower value in minute 5 after birth compared to the non-FIRS group.

**Table 2:** crSO2 (%) in 23 preterm neonates with FIRS and 23 preterm neonates without FIRS (FIRS group and non-FIRS group).

**Time after birth**	**FIRS**	**non-FIRS**	***p*-value**
2 min	31 (23–39)	24 (16–32)	0.234
3 min	36 (28–43)	30 (22–38)	0.301
4 min	40 (33–48)	37 (30–44)	0.526
5 min	44 (37–52)	47 (40–55)	0.559
6 min	51 (44–59)	54 (47–61)	0.629
7 min	57 (50–65)	61 (53–68)	0.569
8 min	62 (55–70)	62 (55–69)	0.949
9 min	67 (59–74)	65 (57–72)	0.696
10 min	71 (64–78)	68 (61–76)	0.593
11 min	72 (65–79)	70 (63–78)	0.749
12 min	72 (64–79)	72 (65–80)	0.903
13 min	71 (63–78)	72 (64–79)	0.820
14 min	71 (64–79)	71 (64–78)	0.921
15 min	73 (65–80)	70 (63–78)	0.648

*Data are presented as mean (95% CI) of the estimated model*.

**Table 3:** cFTOE values in 23 preterm neonates with FIRS and 23 preterm neonates without FIRS (FIRS group and non-FIRS group).

**Time after birth**	**FIRS**	**non-FIRS**	***p*-value**
2 min	0.50 (0.42–0.58)	0.62 (0.54–0.70)	0.037^*^
3 min	0.46 (0.38–0.53)	0.57 (0.50–0.65)	0.032^*^
4 min	0.37 (0.30–0.44)	0.51 (0.44–0.58)	0.006^*^
5 min	0.34 (0.27–0.41)	0.44 (0.37–0.51)	0.050
6 min	0.33 (0.26–0.40)	0.38 (0.31–0.45)	0.322
7 min	0.31 (0.24–0.38)	0.31 (0.24–0.38)	0.983
8 min	0.27 (0.20–0.34)	0.30 (0.23–0.37)	0.610
9 min	0.24 (0.17–0.31)	0.27 (0.20–0.34)	0.531
10 min	0.21 (0.14–0.28)	0.24 (0.17–0.32)	0.531
11 min	0.21 (0.14–0.28)	0.24 (0.17–0.31)	0.486
12 min	0.21 (0.14–0.28)	0.22 (0.15–0.29)	0.819
13 min	0.21 (0.14–0.29)	0.22 (0.15–0.29)	0.863
14 min	0.22 (0.15–0.29)	0.23 (0.15–0.30)	0.914
15 min	0.20 (0.13–0.28)	0.22 (0.15–0.29)	0.773

*Data are presented as mean (95% CI) of the estimated model*.

* *p-value < 0.05*.

### Routine Monitoring

**Figure 4:**
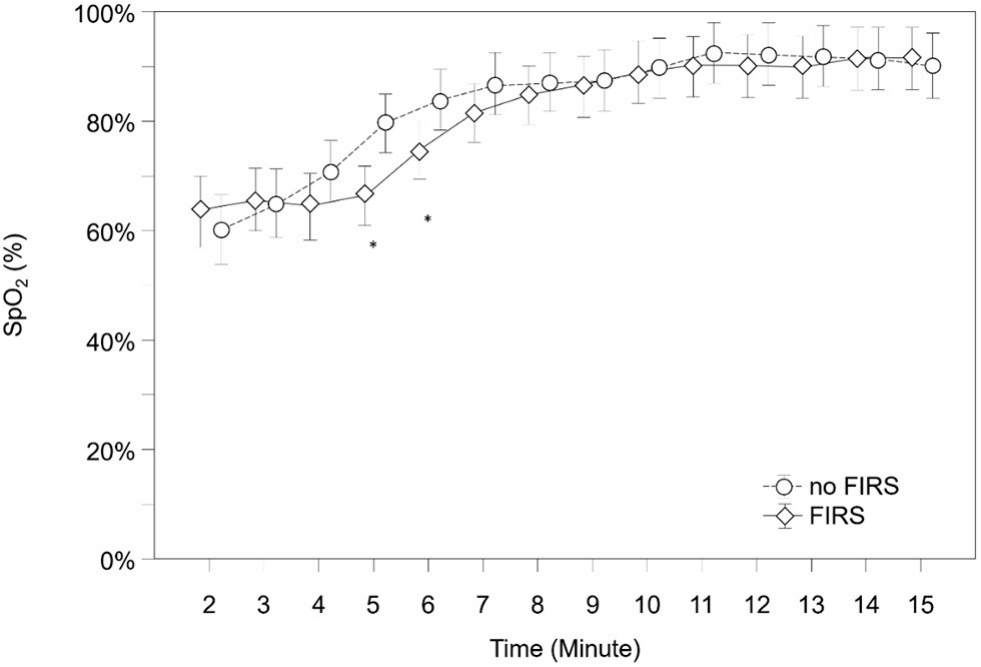
SpO2 in 23 preterm neonates with FIRS and 23 preterm neonates without FIRS (FIRS group and non-FIRS group) during the first 15 min after birth (mean and 95% CI) (*outliers).

**Figure 5:**
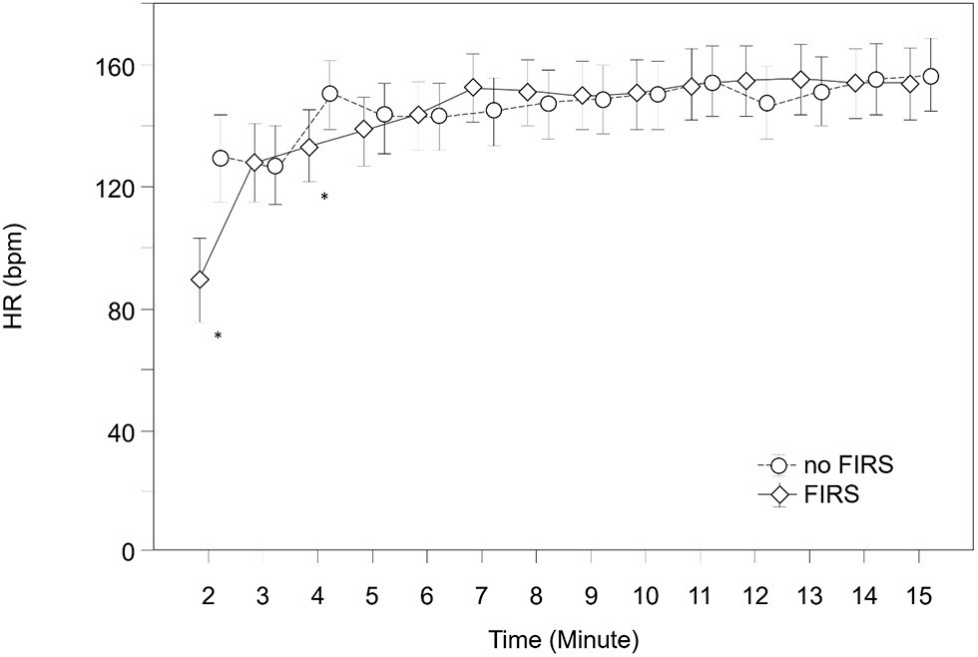
HR in 23 preterm neonates with FIRS and 23 preterm neonates without FIRS (FIRS group and non-FIRS group) during the first 15 min after birth (mean and 95% CI) (*outliers).

**Figure 6:**
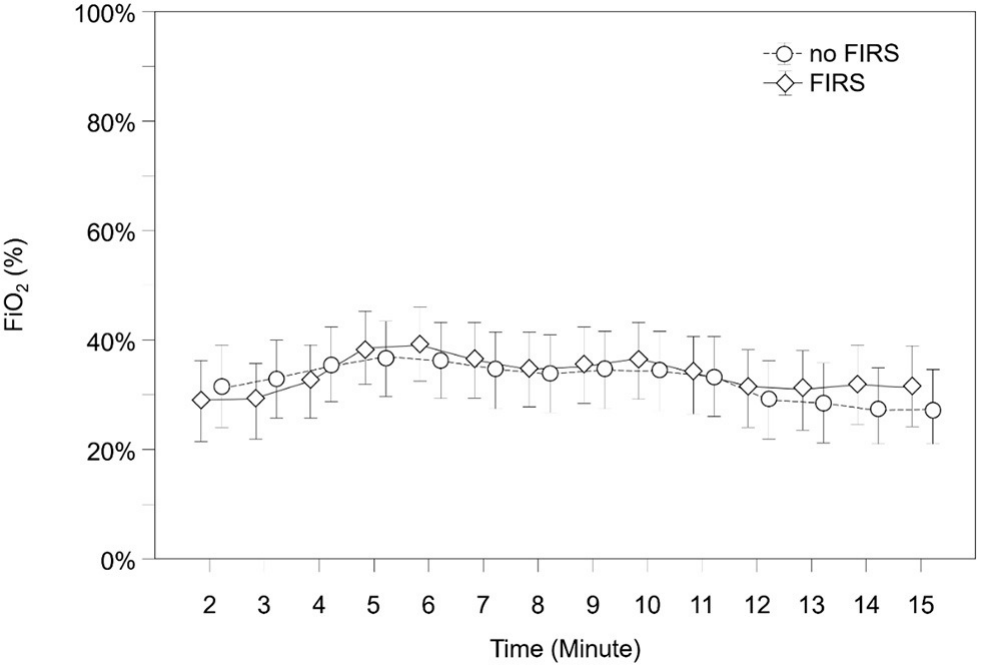
FiO2 in 23 preterm neonates with FIRS and 23 preterm neonates without FIRS (FIRS group and non-FIRS group) during the first 15 min after birth (mean and 95% CI).

SpO2, HR, and FiO2 during the first 15 min after birth are demonstrated in Figures 4–6 and Supplementary Tables 1–3. SpO2 was significantly lower in minutes 5 and 6 after birth in the FIRS group. Afterwards, there were no significant differences between the two groups in SpO2. HR was significantly lower in the FIRS group in minutes 2 and 4 after birth. There were no significant differences in FiO2 between both groups within the first 15 min after birth.

### Cerebral Injury and Mortality

At term-equivalent age or before discharge no significant differences in cerebral injury or mortality were observed. Twenty (87%) preterm neonates in the FIRS group and 22 (96%) neonates in the non-FIRS group survived without cerebral injury. In the FIRS group one neonate had an IVH Grade I, one neonate an IVH Grade III and one neonate a PVL Grade I. The IL6 values of these neonates were 19.7, 686.2, and 959.9 pg/ml, respectively. In the non-FIRS group one neonate had a PVL Grade I (IL-6 value 3.0 pg/ml). One (4%) preterm neonate of the FIRS group died (IL-6 value 959.9 pg/ml).

## Discussion

To our knowledge, this is the first study to evaluate the potential influence of FIRS on cerebral oxygenation measured with NIRS in preterm neonates during the first 15 min after birth. Preterm neonates with FIRS showed significantly lower cFTOE values in the first 4 min after birth compared to neonates in the non-FIRS group.

One would also expect significantly higher crSO2 values when cFTOE is significantly lower. However, in the present study we did not observe significant differences in crSO2 despite significant differences in cFTOE between the groups. This suggests that in the FIRS group not only oxygen consumption but also oxygen delivery was lower, resulting in a similar crSO2 in both groups.

Reduced oxygen consumption during inflammation and sepsis can be explained by mitochondrial dysfunction with impaired energy production (21). There are three possible mechanism for this mitochondrial dysfunction: (i) dysfunction is secondary due to tissue hypoxia, (ii) impairment of oxygen utilization due to cytokines, and (iii) active mitochondrial measure of survival strategy resembling stunning or hibernation. In the present study the first explanation should not play an important role since tissue oxygenation was even higher in neonates with FIRS group.

Cerebral oxygen delivery is a function of Hb concentration, arterial oxygen content and cerebral blood flow depending on cardiac output and vascular resistance. SpO2 representing oxygen content was similar in both groups in the first 4 min. In neonates, cardiac output is strongly dependent on HR. Thus, HR determines strongly cerebral blood flow, depending on vascular resistance (22). HR was significantly lower in the first minutes in the FIRS group compared to the non-FIRS group in the present study. Therefore, it can be assumed that the observed lower HR in the FIRS group resulted in a reduction of cerebral blood flow and thus in a reduction of cerebral oxygen delivery. Cerebral oxygenation and cerebral perfusion might also be influenced immediately after birth by open shunts (persistent ductus arteriosus and persistent foramen ovale) (23). Influence of cardiac output and possible shunts via persistent ductus arteriosus and persistent foramen ovale on the present findings cannot be ruled out completely, since in the included studies no echocardiography was performed. In addition an elevation of IL-6 causes stress due to inflammation, under regulation of neural and endocrine response (24). In unprepared preterm neonates, it is assumed that the production of IL-6 causes stress induced by adrenal gland cells (25). cFTOE measured with NIRS reflects oxygen saturation in veins (70–80%), capillaries (5–10%) and arteries (15–25%) (26). If FIRS resulted in a centralization of the circulation, this may have changed the ratio of the arterial, capillary and venous compartments. As a consequence this resulted in a local decrease of oxygen consumption, because of a higher proportion of arterial blood vessels (relative to reduced number of capillary vessels within the measurement compartments), resulting in higher values of crSO2 and consecutively in lower cFTOE values (27, 28). However, we do not have detailed information about the behavior of cerebral blood flow to define exact changes in cerebral perfusion to prove the underlying mechanism of the observed differences between groups (13, 14, 29).

Rallis et al. (16) measured cerebral oxygenation with NIRS in neonates on the first, third, and seventh day of sepsis. No differences in crSO2 between neonates with sepsis and those without sepsis were found on the first and third day, but crSO2 was severely compromised on the seventh day of sepsis (16). In the present study, we found differences between the FIRS and the non-FIRS group already within the first 5 min after birth, with lower cFTOE values in the FIRS group but no significant differences in crSO2. This inconsistency between the studies can be explained by the different time point of measurement as well as by differences in circulation and ventilation between the first minutes after birth and the first week after birth.

In the present study, there were no differences between the two groups in PCT, determined from umbilical cord blood, and in CRP values, taken on the first and on the second day after birth. Therefore, we assume that FIRS is not always linked with a neonatal infection. Ebenebe et al. (23) investigated factors associated with elevated umbilical cord blood IL-6 values in neonates without infection. They demonstrated that neonates can cope with perinatal stress or intrauterine inflammation (elevated IL-6 values) without developing any clinical signs of inflammation or infection. In detail, out of 471 neonates with no clinical signs of infection within 72 h, 139 neonates showed IL-6 greater 11 pg/ml (25).

In the present study, no significant differences in cerebral injury or mortality at term-equivalent age or before discharge were observed between the two groups, whereby the overall number of cerebral injury and death in both groups was low.

Ozalkaya et al. (30) described that umbilical cord blood IL-6 concentrations > 37.7, > 26.7, and > 17.5 pg/ml predicted death, RDSand multi-organ failure, respectively. In addition, preterm neonates with a gestational age of > 32 weeks and a birth weight > 1,500 g have a lower risk for the development of cerebral injury, like IVH or PVL when compared to neonates born below 30 weeks of gestation (31–34). In our patients, the median gestational age and birth weight were > 32 weeks and > 1,500g, respectively. Further, the majority of IL-6 values in the FIRS group were relatively low. Therefore, we suppose that the published association between FIRS and adverse neonatal outcome (3, 30) may occur more often in preterm neonates with a lower gestational age and birth weight and higher IL-6 values.

### Strengths and Limitations

The strength of the present study is the matching of the FIRS and non-FIRS group, rendering them comparable in terms of demographic parameters. However, due to matching the number of analyzed preterm neonates in both groups became rather small. Furthermore, the majority of IL-6 values in the FIRS group (median IL-6 19.7 pg/ml) was relatively low, although they fulfilled the evidence-based definition of FIRS (1). However, significant differences in cFTOE values between the two groups were observed.

## Conclusion

In preterm neonates with FIRS, crSO2 was similar despite significantly lower cFTOE values during the first minutes after birth compared to neonates without FIRS. This observation may be a result of compromised oxygen consumption and delivery in the first minutes after birth in neonates with FIRS.

## Data Availability Statement

All datasets presented in this study are included in the article/Supplementary Material.

## Ethics Statement

The studies involving human participants were reviewed and approved by Committee on Biomedical Research Ethics, Medicial University of Graz, Auenbruggerplatz 2, 8036 Graz, Austria. Written informed consent to participate in this study was provided by the participants' legal guardian/next of kin.

## Author Contributions

CH, AA, and GP: substantially contributed to the conception and design of the work. CH, MB, NB-S, BS, LM, AA, BU, and GP: analysis and interpretation of data, drafting the work and revising it critically, final approval of the version to be published, and agreement to be accountable for all aspects of the work in ensuring that questions related to the accuracy or integrity of any part of the work are appropriately investigated and resolved.

## Conflict of Interest

The authors declare that the research was conducted in the absence of any commercial or financial relationships that could be construed as a potential conflict of interest.

## Abbreviations

bpm: beats per minute
cFTOE: fractional tissue oxygen extraction
CRP: C-reactive protein
crSO2: cerebral oxygen saturation
FiO2: fraction of inspired oxygen
FIRS: fetal inflammatory response syndrome
HR: heart rate
IL-6: Interleukin-6
IQR: interquartile range
IVH: intraventricular hemorrhage
MABP: mean arterial blood pressure
NIRS: near-infrared spectroscopy
PCT: procalcitonin
PVL: periventricular leukomalacia
SpO2: arterial oxygen saturation.
