# Supplementary material for: Fetal Inflammatory Response Syndrome and Cerebral Oxygenation During Immediate Postnatal Transition in Preterm Neonates
Source: Front Pediatr. 2020 Jul 22;8:401. doi: 10.3389/fped.2020.00401 (PMC7387571; doi:10.3389/fped.2020.00401)
Supplement: Supplementary file 1 [file Table_1.docx]

**Supplementary Table 1.** SpO2 (%) in 23 preterm neonates with FIRS and 23 preterm neonates without FIRS (FIRS group and non-FIRS group). Data are presented as mean (95% CI) of the estimated model.

| Time after birth | FIRS | non-FIRS | p value |
| --- | --- | --- | --- |
| 2 min | 64 (57-70) | 60 (54-67) | .461 |
| 3 min | 66 (60-72) | 65 (59-71) | .846 |
| 4 min | 65 (59-70) | 71 (65-77) | .110 |
| 5 min | 67 (61-72) | 80 (74-85) | .001* |
| 6 min | 75 (69-80) | 84 (79-90) | .022* |
| 7 min | 82 (77-87) | 87 (81-92) | .230 |
| 8 min | 85 (79-90) | 87 (82-93) | .530 |
| 9 min | 86 (81-92) | 88 (82-93) | .762 |
| 10 min | 89 (83-95) | 90 (84-95) | .851 |
| 11 min | 90 (85-96) | 93 (87-98) | .547 |
| 12 min | 90 (85-96) | 92 (87-98) | .592 |
| 13 min | 90 (84-95) | 91 (86-98) | .577 |
| 14 min | 92 (86-97) | 91 (86-96) | .951 |
| 15 min | 92 (86-98) | 90 (85-96) | .747 |

* p-value < 0.05
