# Supplementary material for: Fetal Inflammatory Response Syndrome and Cerebral Oxygenation During Immediate Postnatal Transition in Preterm Neonates
Source: Front Pediatr. 2020 Jul 22;8:401. doi: 10.3389/fped.2020.00401 (PMC7387571; doi:10.3389/fped.2020.00401)
Supplement: Supplementary file 2 [file Table_2.docx]

**Supplementary Table 2.** HR (beats per minute) in 23 preterm neonates with FIRS and 23 preterm neonates without FIRS (FIRS group and non-FIRS group). Data are presented as mean (95% CI) of the estimated model.

| Time after birth | FIRS | non-FIRS | p value |
| --- | --- | --- | --- |
| 2 min | 89 (78-103) | 130 (116-143) | <.001* |
| 3 min | 128 (115-141) | 127 (114-140) | .918 |
| 4 min | 133 (122-145) | 150 (138-162) | .048* |
| 5 min | 139 (127-150) | 143 (132-154) | .586 |
| 6 min | 144 (132-155) | 143 (132-154) | .926 |
| 7 min | 153 (141-164) | 145 (134-156) | .332 |
| 8 min | 151 (140-162) | 147 (136-158) | .599 |
| 9 min | 150 (139-161) | 149 (138-160) | .892 |
| 10 min | 151 (139-162) | 150 (139-162) | .940 |
| 11 min | 154 (142-165) | 155 (143-166) | .884 |
| 12 min | 155 (143-166) | 147 (135-158) | .340 |
| 13 min | 155 (144-166) | 151 (140-163) | .660 |
| 14 min | 154 (143-165) | 155 (144-167) | .863 |
| 15 min | 154 (142-165) | 157 (145-168) | .724 |

* p-value < 0.05
