# Supplementary material for: Fetal Inflammatory Response Syndrome and Cerebral Oxygenation During Immediate Postnatal Transition in Preterm Neonates
Source: Front Pediatr. 2020 Jul 22;8:401. doi: 10.3389/fped.2020.00401 (PMC7387571; doi:10.3389/fped.2020.00401)
Supplement: Supplementary file 3 [file Table_3.docx]

**Supplementary Table 3.** FiO2 values in 23 preterm neonates with FIRS and 23 preterm neonates without FIRS (FIRS group and non-FIRS group). Data are presented as mean (95% CI) of the estimated model.

| Time after birth | FIRS | non-FIRS | p value |
| --- | --- | --- | --- |
| 2 min | 0.29 (0.22-0.36) | 0.32 (0.24-0.39) | .629 |
| 3 min | 0.29 (0.22-0.36) | 0.33 (0.26-0.40) | .436 |
| 4 min | 0.33 (0.26-0.39) | 0.36 (0.29-0.43) | .534 |
| 5 min | 0.39 (0.32-0.45) | 0.37 (0.30-0.44) | .683 |
| 6 min | 0.40 (0.33-0.46) | 0.36 (0.29-0.43) | .519 |
| 7 min | 0.36 (0.29-0.43) | 0.35 (0.28-0.42) | .735 |
| 8 min | 0.35 (0.28-0.42) | 0.34 (0.27-0.41) | .849 |
| 9 min | 0.36 (0.29-0.43) | 0.35 (0.28-0.42) | .849 |
| 10 min | 0.36 (0.29-0.44) | 0.34 (0.27-0.42) | .693 |
| 11 min | 0.34 (0.27-0.41) | 0.34 (0.26-0.41) | .963 |
| 12 min | 0.31 (0.24-0.38) | 0.29 (0.22-0.37) | .699 |
| 13 min | 0.31 (0.24-0.38) | 0.28 (0.21-0.36) | .637 |
| 14 min | 0.32 (0.25-0.39) | 0.27 (0.21-0.35) | .389 |
| 15 min | 0.32 (0.24-0.39) | 0.27 (0.21-0.35) | .425 |
